# Efficacy of the insect growth regulator novaluron in the control of dengue vector mosquitoes *Aedes aegypti* and *Ae. albopictus*

**DOI:** 10.1038/s41598-024-52384-x

**Published:** 2024-01-23

**Authors:** J. M. Manel K. Herath, W. A. Priyanka P. De Silva, Thilini C. Weeraratne, S. H. P. Parakrama Karunaratne

**Affiliations:** 1 Entomological Surveillance Unit, Office of Regional Director of Health Services, Kurunegala, Sri Lanka; 2 Postgraduate Institute of Science, University of Peradeniya, Peradeniya, Sri Lanka; 3 Department of Zoology, Faculty of Science, University of Peradeniya, Peradeniya, Sri Lanka

**Keywords:** Diseases, Health care

## Abstract

Insect Growth Regulator (IGR) novaluron is an alternative to synthetic neuro-inhibitory insecticides. Present study was designed to assess appropriate dosages of novaluron for dengue vector control. Larvae of *Aedes aegypti* and *Ae. albopictus* were exposed to a concentration series of novaluron (Rimon EC10) for two fixed exposure periods of 7-days and 14-days to determined LC_50_ and LC_99_ values. Inhibition of adult emergence (IE_50_ and IE_99_) was determined by a 14-day exposure. Semi-field experiments were conducted by exposing cohorts of *Ae. aegypti* larvae to IE_99_, 2 × IE_99_ and 10 × IE_99_ novaluron concentrations in water storage buckets (10 L) and plastic barrels (200 L). For the 7-day exposure, LC_50_ values were 0.047–0.049 ppm and LC_99_ were 0.144–0.151 ppm. For 14-day exposure, these values were 0.002–0.005 ppm and 0.006–0.01 ppm respectively. For both species, IE_99_ was 0.001 ppb under semi-field conditions, and was effective for nearly 2 months. Novaluron concentration 0.01 ppb was effective up to 3 months, with an IE of 89–95%. Authorities should critically review a reduction of the presently recommended field dosage of 200 ppm novaluron by × 100 or more. This would provide the same efficacy but mitigate environmental pollution, development of vector resistance, and financial losses.

## Introduction

Dengue fever is a mosquito-borne tropical disease caused by the dengue virus. It has been estimated that 3.9 billion people in the world are at risk of the disease. In Sri Lanka, dengue fever (DF) has become a serious public health concern especially with the alarming increase of cases in the recent past (Epidemiology reports^1^. The disease is transmitted through the bites of the mosquitoes *Aedes aegypt*i and *Ae. albopictus*^2^. Two transmission periods of the disease in Sri Lanka is parallel to the two monsoon seasons, showing the close association between dengue transmission and the rain fall. The most intensive transmission period June to August coincides with the southwest monsoon, whereas the less intense October to December period coincides with the northeast monsoon^3,4^.

Vector control plays the major role in reducing the disease burden especially in the absence of an effective vaccine against dengue. Suppression of vector densities is commonly attempted through various methodologies such as use of insecticides, source reduction, and implementation of new regulations for vector elimination^5^. Although the general public is urged to destroy or remove all possible indoor and outdoor *Aedes* breeding sites, it has not always been effective due to inadequacy of resources and poor community support^6^. Use of insecticides has emerged as a predominant and efficient approach in the control of *Aedes* vector populations, particularly during disease outbreaks^7^. Among the WHO recommended insecticides, pyrethroids with low mammalian toxicity and a higher efficacy are widely used in vector control programmes^5,8^. For more than two decades, Sri Lanka has been using pyrethroids as adulticides and temephos (organophosphate) as a larvicide for *Aedes* mosquito control. Constant application and indiscriminate use have led to emergence of insecticide resistance in *Aedes* populations. Several studies have reported high incidence of resistance to neuroinhibitory insecticides in Sri Lankan dengue vectors^9–15^. Also, the application of temephos as a larvicide is especially a concern due to its toxic effect on non-target aquatic life^16,17^. Furthermore, research has shown that diversifying control methods, such as incorporating biological control agents^18^ or insect growth regulators^19^ can provide sustainable and long-term solutions for managing dengue vector populations while reducing dependence on neuroinhibitory insecticides. Therefore dengue vector control programmes are looking for effective alternatives to minimize the use of neuroinhibitory insecticides.

Insect growth regulators (IGR) have been introduced as potential alternatives to control dengue vector populations^20^. Among IGRs, novaluron is considered as an active agent for insect larval control worldwide (WHO, 2007^21^). This benzoylphenyl urea compound affects larval and pupal stages of the Orders Coleoptera, Diptera and Hemiptera^22–25^. Novaluron has been successfully used to control *Aedes* mosquito larvae^26^. Although the mechanism of action of novaluron has not been extensively investigated, the general mechanism of benzoylphenyl urea action has been well documented^27–29^. Benzoylphenyl urea changes the elasticity and rigidity of the endocuticle in insect immature stages by changing cuticular composition. This mainly affects molting stages of insects, causing death by abnormal endocuticular deposition and interrupted molting^28^. At sub lethal dosages, novaluron has a low risk to the environment, including its effect to mammals, birds and aquatic insect species other than mosquito larvae^30,31^.

Novaluron has been introduced to dengue vector control programs in many countries as an alternative to neuroinhibitory insecticides. However, there are still unanswered questions regarding its optimum field dosage. Although the WHO recommended discriminating dosage for novaluron is 10–50 ppb for dengue vector species^25^, many researchers, after evaluating the efficacy of the compound, have concluded different effective concentrations for field applications based on their findings^26,32–35^. It has been observed that novaluron acts as an emergence inhibitor at lower dosages but as a larvicide at higher concentrations. However, the LC_50_, LC_90_ and EI 100% concentration are inconsistent among the published literature. Although the application of higher dosages results larval death^32^, it has a negative impact on the environment and non-target organisms^36,37^. Dosages which inhibit larval emergence may be sufficient for the strategy to control the vector mosquitoes. Therefore, a proper understanding on larvicidal and emergence inhibition dosages of novaluron is needed for better usage of this IGR in vector control programmes. The present study was designed to undertake laboratory and semi-field trials to evaluate the efficacy and residual effect of novaluron (Rimon EC [emulsifiable concentrate] against immature stages of *Ae. aegypti* and *Ae. albopictus*.

## Methods

### Mosquito larvae and novaluron

During the period of January 2020 to April 2020, mosquito eggs were collected using ovitraps from Bandaranayakepura in Kurunegala Medical Officer of Health (MOH) area, where novaluron had not been used for vector control programs. Collected mosquito eggs were brought to the laboratory of Entomological Surveillance Unit, Office of the Regional Director of Kurunegala and transferred to hatching trays where they were allowed to hatch. Third instar larvae were used for all bioassay experiments with novaluron. Formulated emulsifiable novaluron (100 g/L, Rimon EC10) was a gift from Makhteshim Chemical Works Ltd, Israel.

### Bioassay experiments

#### Determination of LC_50_ and LC_99_ values against novaluron

Novaluron bioassays were performed according to the guidelines of WHO^5^. Stock solution (10,000 ppm) of novaluron was prepared in tap water by 10 × dilution of 100 g/L, Rimon EC10 formulated novaluron. Desired concentrations of novaluron for the bioassay experiments were prepared by mixing the calculated volumes of the stock solution and tap water. Batches of 25 third instar larvae of *Ae. aegypti* and *Ae. albopictus* were exposed separately to eight concentrations (i.e. 0.0001 ppm, 0.001 ppm, 0.01 ppm, 0.05 ppm, 1 ppm, 2 ppm, 3 ppm, 4 ppm) of novaluron. A control experiment was conducted along with each experiment using tap water alone. Assay with each novaluron concentration was replicated 4 times. The number of dead immature stages and the number emerged as adults were counted at 24-h intervals during a 14-day period. Mortality data, obtained for a minimum of four concentrations giving mortality between 0 and 100%, at the end of 7th day (0.0001 ppm–0.05 ppm) or at the end of the 14th day (0.00001 ppm–0.05 ppm) were used separately for probit-regression analysis using SPSS to determine LC_50_ and LC_99_ values. All bioassays were undertaken at the insectary under controlled conditions 26 ± 2 °C and 80 ± 5% RH.

#### Determination of IE_50_ and IE_99_ values against novaluron

Insectary-reared 25 third instar larvae of *Ae. aegypti* and *Ae. albopictus* were exposed separately to concentrations in the range 0.00005 ppb-0.005 ppb novaluron. Adult emergence was assessed by exuviae counts and corresponding flying adults for a period of 14 days. The effect of novaluron was expressed in terms of percentage inhibition of adult emergence (IE %) which is the percentage of larvae that do not develop successfully into emerging adults compared to the control^5^. In recording IE% for each concentration, moribund and dead larvae and pupae, and partial adults that could not completely separate from the pupal cases, were considered as “affected”. Four concentrations giving IE% between 0 and 100% were used for probit-regression analysis using SPSS to determine IE_50_ and IE_99_ values for one week period and for 2-week period separately.

#### Semi-field trials

Residual effect of novaluron on *Ae. aegypti* 3rd instar larvae under semi-field conditions were assessed according to procedure described by WHO^24,25^ and WHOPE^38^, in two different types of water storage containers,water storage buckets (10 L and plastic barrels (200 L, the two most popular forms of water-storage containers used by house holders in the area. Aliquots of novaluron stock solution were added to the test containers with tap water to obtain required concentrations. Novaluron dosage 0.001 ppb, which gave IE_99_ for a 14-day period during the laboratory experiments, was used as the standard and, water storage buckets and plastic barrels were prepared with three dosages; 0.001 ppb (IE_99_), 0.002 ppb (2 × IE_99_) and 0.01 ppb (10 × IE_99_). Sentinel cages were used to place larvae (25 third instar larvae in each cage) in each water storage bucket/plastic barrel. This facilitated the observations on inhibition of emergence and mortality counts.

Sentinel cages were prepared by using 250 mL transparent plastic cups. Bottom of each cup was replaced with a plastic mosquito mesh. After placing larvae, the cups were covered with lids prepared by the same mesh. Each cup was emersed in water in the bucket or the barrel and its positions was stabilized using a wire linked to barrel/ bucket rim. After adding larvae to a sentinel cup, the water storage container was covered by a net to prevent mosquito egg laying. Control experiments were conducted without novaluron. Experimental set up included thirteen buckets (three test concentrations each with 4 replicates, and a control) and thirteen barrels, and were placed in an outdoor undisturbed area with a covered roof. Larval food was added once a week and water loss was replenished weekly.

A larval cohort (batch of 25) was introduced to the sentinel cage in the fresh novaluron solution filled bucket/barrel at the beginning of each experiment. After a 7-day exposure period, larvae of the first cohort were removed and a second cohort was introduced. Third cohort replaced the second cohort after another 7 days. Thereafter, each cohort was replaced by a new cohort once in a 14-day period. Before introducing a new cohort (batch of 25), all the dead/survive larvae from the previous cohort were removed. Assessment of larval survivorship was recorded. Adult emergence was assessed by counting pupal skins and emerged adults. After the count, remaining pupal skins were removed by a syringe or a fish net before the next cohort was introduced. Observations and recordings were continued until all the immature stages in the control emerged into adults.

### Data analysis

Dose response curves were generated by using cumulative mortality in each concentration within the specific time period. Percentage of inhibition of emergence (IE%) was calculated using the following equation.

$$\% {\text{IE}} = 100 - \left( {{\text{T}}/{\text{C}}*100} \right)$$

where T is the number of emerged adults in tested units, C is the number of emerged adults in control unit^5,26, 39^.

Mortality and IE data obtained at each concentration was subjected to probit-regression analysis to determine LC_50_ and LC_90_ values and, IE_50_ and IE_99_ values using SPSS (v.9.0) program for Windows.

Residual effect in semi-field data was analyzed by one-way ANOVA. Kruskal–Wallis test was employed to compare adult emergence inhibition and adult emergence rates across all stand-alone and combination treatments. This non-parametric test was well-suited for comparing medians, particularly in cases where a non-normal distribution was observed, a condition not accommodated by the ANOVA test. A significance level of *p* < 0.05 was considered statistically significant.

## Results

**Figure 1:**
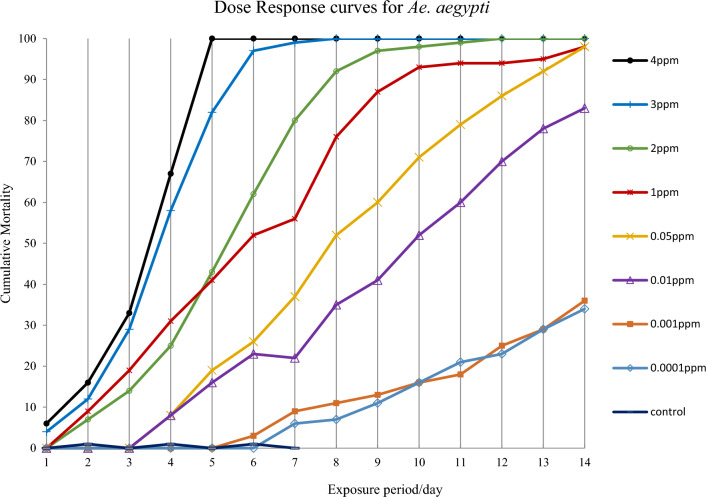
Cumulative mortality of immature stages of *Aedes aegypti* after exposing 3rd instar larvae to 0.0001 ppm–4 ppm novaluron concentrations under laboratory conditions (n = 100 for each concentration).

**Figure 2:**
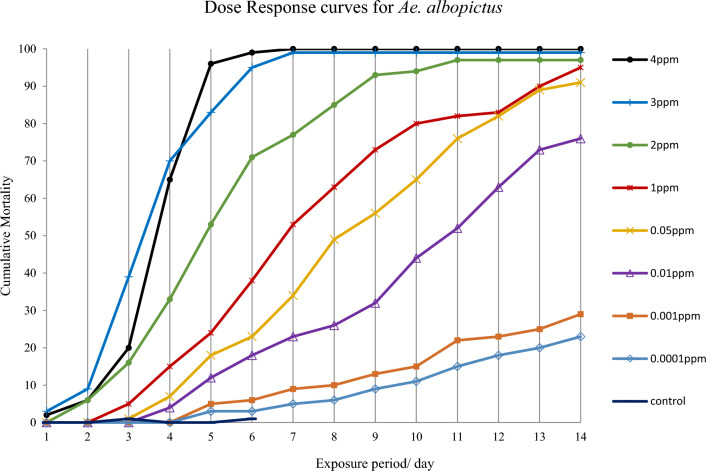
Cumulative mortality of immature stages of *Aedes albopictus* after exposing 3rd instar larvae to 0.0001 ppm–4 ppm novaluron concentrations under laboratory conditions (n = 100 for each concentration).

At the end of the 7-day period, larvae in control groups exhibited 100% emergence rate. The cumulative mortality of *Ae. aegypti* and *Ae. albopictus* 3rd instar larvae treated with eight different concentrations of novaluron under laboratory conditions are shown in Fig. 1. At the highest concentrations used (3 ppm and 4 ppm) *Ae. aegypti* larvae started to die within the first 24-h exposure whereas at the lowest concentration (0.0001 ppm) the mortality started only after 7 days. The highest concentration 4 ppm gave 100% mortality after 5 days of exposure and the concentrations 1.0 ppm and below did not result 100% mortality even after 14 days of exposure. Results showed that *Ae. albopictus* is slightly more resistant to novaluron. During a 24-h exposure period, *Ae. albopictus* larval mortalities were 3% and 10% lower than *Ae. aegypti* larval mortalities at the concentrations 3 ppm and 4 ppm, respectively. Concentrations 2 ppm and below could not achive 100% mortality at the end of the 14 days period (Figs. 1 and 2).

**Figure 3:**
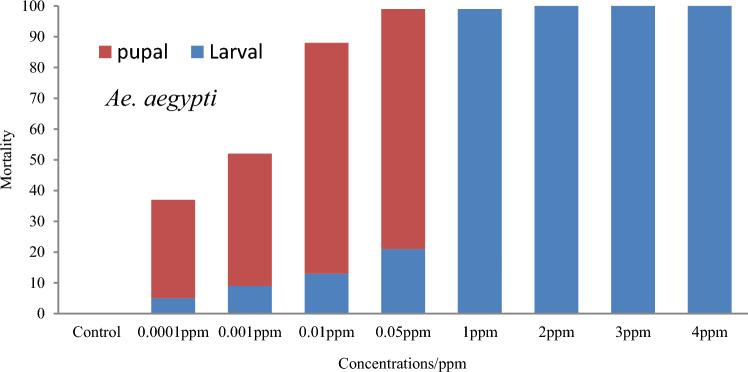
Stage-specific mortality of *Aedes aegypti* after exposing 3rd instar larvae to 0.0001 ppm–4 ppm novaluron concentrations for a period of 14 days (n = 100 for each concentration).

**Figure 4:**
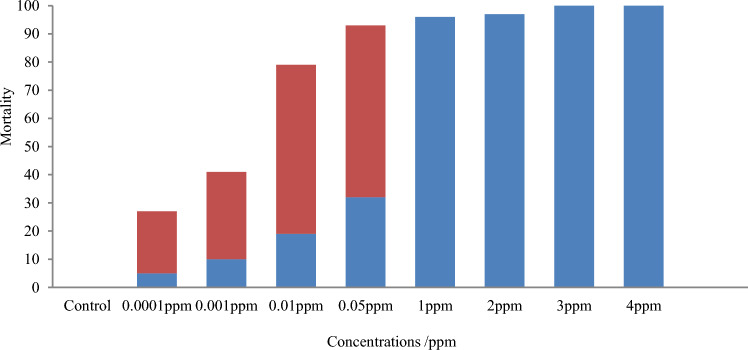
Stage-specific mortality of *Aedes albopictus* after exposing 3rd instar larvae to 0.0001 ppm–4 ppm novaluron concentrations for a period of 14 days (n = 100 for each concentration).

During the 7 days and 14 days exposure period, pupation of larvae of both species occurred only in the concentrations 0.05 ppm and below. At these concentrations, deformities could be observed both in larvae (elongated thorax with pigmentation and with a capsuled head) and pupae (incomplete molting resulting intermediate stage between larva and pupa). In the concentrations above 1.0 ppm, larval mortality without pupation occurred (Figs. 3 and 4). *Aedes aegypti* showed a statistically significant (*p* = 0.002, F = 10.78) higher pupal mortality compared to that of *Ae. albopictus*.

Lethal concentrations of novaluron which killed 50% (LC_50_) and 99% (LC_99_) of larvae/pupae at the end of 7-day period and at the end of 14-day periods and the calculated IE_50_ and IE_99_values for 14-day exposure periods are presented in the Table 1.

**Table 1:** LC_50_ and LC_99_ values of novaluron for *Aedes aegypti* and *Ae. albopictus* larvae for 7-day and 14-day exposure periods and, IE50 and IE99 values for 14 day exposure period (n = 100 for each species per exposure period).

Species	Exposure period (days)	LC_50_ ppm (95% CI)	LC_99_ ppm (95% CI)	IE _50_ ppb	IE _99_ ppb
*Ae. aegypti*	7	0.047 (0.007–0.055)	0.144 (0.01–0.150)		
	14	0.002 (0.001–0.002)	0.006 (0.002–0.008)	0.0003	0.0009
*Ae. albopictus*	7	0.049 (0.006–0.081)	0.151 (0.146–0.184)		
	14	0.005 (0.001–0.003)	0.01 (0.012–0.08)	0.0004	0.001

When the LC_50_ values for 7-day and 14-day exposure periods are compared, concentrations needed to kill 50% or 99% of *Ae. aegypti* population in 7 days were about 24 times higher than the respective concentrations needed for the 14 day period. For *Ae. albopictus*, the respective concentrations needed were about 10–15 times higher (Table 1).

**Figure 5:**
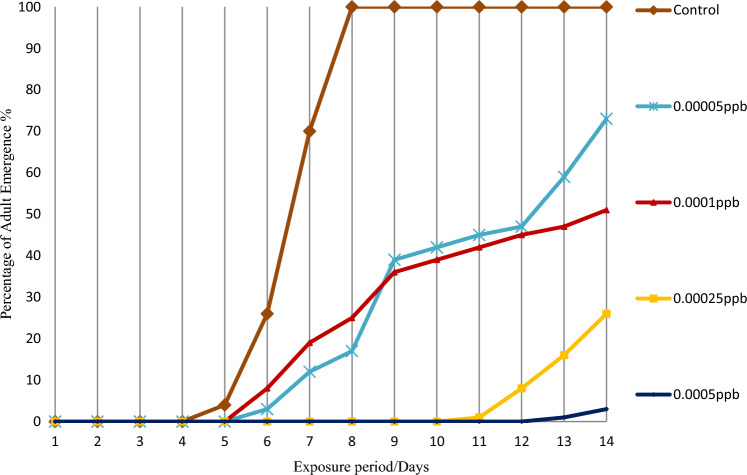
Adult emergence percentage of *Aedes aegypti*, after exposing 3rd instar larvae to 0.00005 ppb–0.0005 ppb novaluron concentrations under laboratory conditions (n = 100 for each concentration).

**Figure 6:**
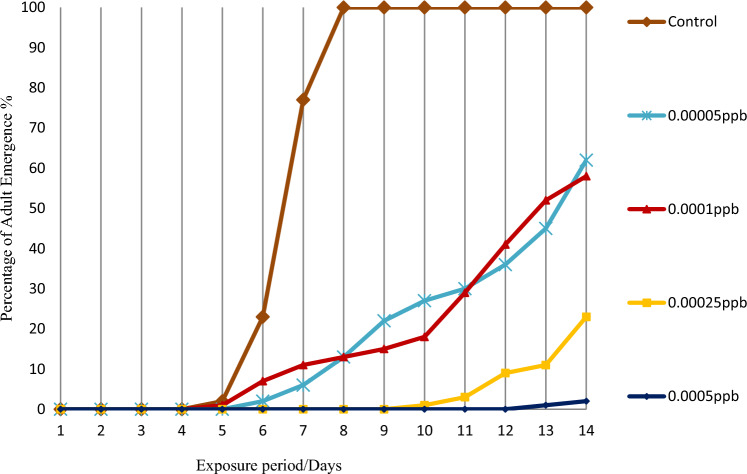
Adult emergence percentage of *Aedes albopictus*, after exposing 3rd instar larvae to 0.00005 ppb–0.0005 ppb novaluron concentrations under laboratory conditions (n = 100 for each concentration).

Adult emergence percentage by novaluron during the 14-day period is shown in the Figs. 5 and 6. In the controls, adult emergence of both species started from 5th day onwards. *Aedes aegypti* larvae started to emerge after the 6th day of exposure at the lowest 0.00005 ppb concentration and after 13 days of exposure at the highest 0.005 ppb concentration. After 14 days, adult emergence was 73 ± 0.12 at 0.00005 ppb and 3 ± 0.53 at 0.0005 ppb (Fig. 5). Corresponding values for *Ae. albopictus* were 62 ± 0.4 and 2 ± 0.2 respectively, showing that adult emergence inhibition for *Ae. albopictus* was more or less similar to that of *Ae. aegypti* for the tested novaluron concentrations (Fig. 6).

All three novaluron concentrations used for the semi-field experiments caused 96% adult emergence inhibition up to 2 weeks and 88–100% IE up to 4 weeks (one month) (Table 2). An inhibition of 55%-64% was observed at 0.001 ppb–0.002 ppb concentrations up to a 7-week period. Novaluron concentration 0.01 ppb was highly effective giving 89–95% IE for a 3-month and 67–68% IE for a 4-month period. No significant differences were observed in IE values (F = 0.09, *p* < 0.05) and adult emergence (F- 0.087, *P* < 0.05) between the two types of containers tested.

**Table 2:** Emergence inhibition (IE) of *Aedes aegypti* larvae exposed to novaluron under semi-field conditions.

Cohort number	Age of the treatment (days)	Control IE%		0.001 ppb IE%		0.002 ppb IE%		0.01 ppb IE%	
		WS-buckets	WS-barrel	WS-buckets	WS-barrel	WS-buckets	WS-barrel	WS-buckets	WS-barrel
1	7	0	0	100	100	100	100	100	100
2	14	0	0	97	98	99	96	100	100
3	28	0	0	96	95	97	90	100	99
4	42	0	0	91	90	98	88	96	98
5	56	0	0	81	87	86	83	98	94
6	70	0	0	77	76	89	79	96	90
7	84	0	0	55	64	63	58	95	89
8	98	0	0	3	13	16	13	77	72
9	112	0	0	0	2	1	2	67	68

Larval cohorts (25 larvae per cohort) were exposed in water storage (WS) buckets and barrels. Cohorts were replaced once in 7 days (first two cohorts) or in 14 days (n = 100 per concentration per exposure).

## Discussion

Although dengue infections have been documented from Sri Lanka since the 1960s, the first significant DHF outbreak with 206 clinically diagnosed cases and 20 deaths (CFR 9.8%) occurred in Colombo in 1989^40^. Since then, dengue outbreaks have been gradually spreading to many regions of the island, with an increase in reported cases occurring every 3 to 5 years (Ministry of Health Sri^41^. Increasing tendency of dengue prevalence in Sri Lanka emphasizes the urgent need of effective vector control measures. Traditional vector control methods have several limitations including vector resistance^15,42, 43^. Biological insecticides based on *Bacillus thuringiensis* (*Bt*) have been employed in Sri Lanka as an alternative in integrated vector control strategies with varying success^42^. Storage challenges and cost-effectiveness, and resistance development are major challenges against *Bt* insecticides^26,44, 45^. Insect growth regulators (IGRs) are a group of chemicals that have been recently used as an effective alternative for controlling insect vectors worldwide^7^. They exhibit a good margin of safety to most non-target biota, thus offering some advantages in mosquito control programmes^26^ and are strictly arthropod-specific and environmentally safe and show very low toxicity to birds, mammals and honey bees^24,46^. Novaluron 10 EC formulation has been developed under WHOPES guidance and supervision, as an IGR against insects, including mosquitoes^24,25^.

Previous studies have shown the efficacy of novaluron against *Ae. aegypti* and *Ae. albopictus*^28^, Arredondo-Jiménez et al.^35^, *Culex quinquefasciatus*^26^, Arredondo-Jiménez et al.^35^ and *Anopheles* species^47^. Novaluron is reportedly effective against *Aedes* larvae, although it is unclear what dosages are to be applied in vector control programs.

Present results shows that *Ae. albopictus* slightly more resistant to novaluron than *Ae. aegypti*, an observation made by some previous workers as well^33,35^. Both vector species exhibit 100% larval mortality within a 24-h exposure period at 3 ppm–4 ppm novaluron concentrations, which is lower than the 9.43 ppm dosage reported for the same purpose by Gunathilaka et al.^32^ for *Aedes aegypti*. Also, our results revealed that, in a 14-day exposure, a concentration of 1.0 ppm is enough to kill both *Ae. aegypti* and *Ae. albopictus* larvae before pupation. Even at 0.05 ppm, although some larvae pupated to deformed pupae, all larvae and pupae got killed during the 14-day exposure. Deformities caused by abnormal endocuticular deposition and interrupted molting are expected from novaluron exposure^28^. Similar deformed pupations had been observed by Mulla et al.^26^ at 0.25 ppb technical novaluron concentration for the 2nd instar *Ae. aegypti* larvae and by Fontoura et al.^34^ at 0.40 ppb formulated novaluron concentration for 3rd instar *Ae. aegypti* larvae, under field conditions.

Lethal concentrations of novaluron which kill 50% (LC_50_) and 99% (LC_99_) of larvae/pupae for 7-day period exposure was 0.047 ppm and 0.144 ppm respectively for *Ae. aegypti*, and 0.049 ppm and 0.151 ppm for *Ae. albopictus* respectively. Arredondo-Jiménez et al.^35^ reported LC_50_ and LC_99_ values 0.025 ppm and 0.07 ppm respectively for *Ae. aegypti* and, 0.035 ppm and 0.09 respectively for *Ae. albopictus* for the 7-day period exposure to the same formulated novaluron (Rimon 10EC). For a 24-h exposure, Gunathilaka et al.^32^ have reported 2.72 ppm LC_50_ and 9.43 ppm LC_99_ for *Ae. aegypti.* For a 14-day exposure period, Withanage et al.^33^ have reported × 10 lower LC_50_ and LC_99_ values (0.0002 ppm and 0.01 ppm respectively for *Ae. aegypti*, 0.0003 ppm and 0.004 ppm respectively for *Ae. albopictus*) than ours for the same formulated novaluron.

Novaluron, which is an insect growth regulator, inhibits adult emergence and the immature stages ultimately die after a prolonged immature period. We could observe this effect of novaluron clearly at lower concentrations. Under laboratory conditions, 50% adult emergence inhibition came from 0.0003 ppb for *Ae. aegypti* and from 0.0004 ppb for *Ae. albopictus* whereas 99% adult emergence inhibition was achieved by both species at 0.001 ppb. Present study indicated a higher efficacy of novaluron than that has been reported previously i.e., 550 ppb IE_99_ for both vectors^35^, 0.14 ppb IE_50_ and 0.22 ppb IE_99_^34^ and, 0.09 ppb IE_50_ and 0.1 ppb IE_99_ for *Ae. aegypti*^48^ for a 14-day exposure period.

During our study, residual effect of novaluron was evaluated under semi-field conditions. Number of days during which the emergence inhibition is more than 80% is considered as the duration of effectiveness^38^. According to this definition, even 0.001 ppb was effective nearly for 2 months. Novaluron concentration 0.01 ppb was effective for 3 months, with an IE of 89–95%. Fontoura et al.^34^ and Arredondo-Jiménez et al.^35^ have reported that formulated novaluron (Rimon 10EC) is effective for a 4 month period at concentrations 0.22 ppb and 0.55 ppm respectively which are nearly × 10 and × 10,000 times respectively higher than the 0.01 ppb concentration we demonstrated for 3 months.

Our data demonstrates that 0.02 ppm concentration of formulated novaluron (Rimon 10EC) can be effectively used as a larvicide against dengue vectors. It will kill larvae before pupation. A concentration 0.01 ppb is effective for 3 months as an adult emergence inhibitor. However, the present WHO recommended dosage is 0.01–0.05 ppm^24,25^. Gunathilaka et al.^32^ have recommended 20 ppm dosage of novaluron for vector control against Sri Lankan *Aedes* mosquitoes. This concentration gave 100% mortality within 24 h of application. In Sri Lanka, the label of the commercially available Rimon EC10 bottle states that the recommended dosage to be used for larval breeding sites is 200 ppm which is an extraordinarily high value. Authorities should take this matter seriously and reduce the recommended dosages for field application to an appropriate level considering scientific data coming from research studies so that environmental pollution, effect on non-target insect larvae, resistance development and financial loss can be minimized.

Our study highlights that novaluron can contribute significantly to vector control strategies by offering an effective, environment friendly and a cost-effective alternative to traditional insecticides. Also, our results challenge the current guidelines on novaluron usage, advocating for lower dosages. The work presented here was limited to laboratory and semi-field conditions. Field studies to validate these findings in real-world settings are needed to confirm the findings. Also, long-term impact on mosquito populations and potential resistance development and management, which are crucial for assessing the sustainability of this vector control strategy, require further research. A broader environmental impact analysis to fully validate and support the argument for changing current vector control practices is also a necessity.

## Conclusion

Formulated novaluron (Rimon 10EC) 0.02 ppm concentration can be effectively used as a larvicide against dengue vectors. A concentration 0.01 ppb is effective for 3 months as an adult emergence inhibitor. The recommended dosages for the field application of novaluron 200 ppm is extraordinarily high, and the authorities should reconsider their recommendation to minimize environmental pollution, the effect on non-target insect larvae, resistance development, and financial loss.

## Data Availability

The data used to support the findings of this study are available from the corresponding author upon request.
